# Plant-Dependent Soil Bacterial Responses Following Amendment With a Multispecies Microbial Biostimulant Compared to Rock Mineral and Chemical Fertilizers

**DOI:** 10.3389/fpls.2020.550169

**Published:** 2021-02-04

**Authors:** Bede S. Mickan, Ahmed R. Alsharmani, Zakaria M. Solaiman, Matthias Leopold, Lynette K. Abbott

**Affiliations:** ^1^UWA School of Agriculture and Environment (M079), The University of Western Australia, Perth, WA, Australia; ^2^UWA Institute of Agriculture (M082), The University of Western Australia, Perth, WA, Australia; ^3^College of Science, University of Kufa, Najaf, Iraq

**Keywords:** biostimulants, soil bacteria, microbial inoculant, pasture, soil biology

## Abstract

Biostimulants are gaining momentum as potential soil amendments to increase plant health and productivity. Plant growth responses to some biostimulants and poorly soluble fertilizers could increase soil microbial diversity and provide greater plant access to less soluble nutrients. We assessed an agricultural soil amended with a multispecies microbial biostimulant in comparison with two fertilizers that differed in elemental solubilities to identify effects on soil bacterial communities associated with two annual pasture species (subterranean clover and Wimmera ryegrass). The treatments applied were: a multispecies microbial biostimulant, a poorly soluble rock mineral fertilizer at a rate of 5.6 kg P ha^–1^, a chemical fertilizer at a rate of 5.6 kg P ha^–1^, and a negative control with no fertilizer or microbial biostimulant. The two annual pasture species were grown separately for 10 weeks in a glasshouse with soil maintained at 70% of field capacity. Soil bacteria were studied using 16S rRNA with 27F and 519R bacterial primers on the Mi-seq platform. The microbial biostimulant had no effect on growth of either of the pasture species. However, it did influence soil biodiversity in a way that was dependent on the plant species. While application of the fertilizers increased plant growth, they were both associated with the lowest diversity of the soil bacterial community based on Fisher and Inverse Simpson indices. Additionally, these responses were plant-dependent; soil bacterial richness was highly correlated with soil pH for subterranean clover but not for Wimmera ryegrass. Soil bacterial richness was lowest following application of each fertilizer when subterranean clover was grown. In contrast, for Wimmera ryegrass, soil bacterial richness was lowest for the control and rock mineral fertilizer. Beta diversity at the bacterial OTU level of resolution by permanova demonstrated a significant impact of soil amendments, plant species and an interaction between plant type and soil amendments. This experiment highlights the complexity of how soil amendments, including microbial biostimulants, may influence soil bacterial communities associated with different plant species, and shows that caution is required when linking soil biodiversity to plant growth. In this case, the microbial biostimulant influenced soil biodiversity without influencing plant growth.

## Introduction

In grassland systems where the aim is to promote plant biomass, application of fertilizers is the most common and important management practice (Ikoyi et al., 2018; Carswell et al., 2019). However, the use of biostimulants to compliment fertilizers is gaining interest (Caradonia et al., 2019). Plant growth responses to some biostimulants could influence the soil microbial community and provide greater plant access to less soluble nutrients (Calvo et al., 2014). Biostimulants include multispecies microbial inoculants and may be used alone (e.g., Assainar et al., 2018) or in combination with fertilizers (e.g., Assainar et al., 2020).

The success of microbial inoculants as biostimulants is varied and may not be predictable (Qiu et al., 2019). For example, in previous studies of the use of a multispecies microbial inoculant applied to wheat, there was a positive response in grain yield (Assainar et al., 2018). The microbial inoculant influenced the relative abundance of rhizosphere bacteria, especially Actinobacteria. However, in another study using the same multispecies microbial inoculant, there was no benefit from the introduction of the multispecies microbial inoculant in terms of fertilizer use efficiency for wheat (Assainar et al., 2020). Despite the rapid expansion of interest in commercial use of microbial inoculants (Qiu et al., 2019; Sammauria et al., 2020), further investigation is required to assist farmers discriminate among management practices that involve microbial products as biostimulants in terms of their efficacy (Abbott et al., 2018).

Conventional P fertilizers promote pasture growth but they can be expensive, especially in developing regions (Sanchez, 2002) and may have adverse influences on the environment and on soil microbial communities (Pan et al., 2014; Luo et al., 2015; Ikoyi et al., 2018). Thus, alternative fertilizers such as controlled-release fertilizers or slow-released fertilizers have been used to minimize the negative consequences of supplying unnecessarily high levels of soluble P (Hagin and Harrison, 1993; Shaviv, 2001; Van Geel et al., 2016). Slow-release fertilizers have been defined as classes of fertilizers that contain moderately soluble components regardless of the properties of the reaction products in the soil (Hagin and Harrison, 1993). The release pattern of P is important to control the concentration of phosphate ions in the soil solution and to minimize environmental loss (von Sperber et al., 2017; Fischer et al., 2018).

A range of fertilizers, including organic P-based fertilizers, can regulate the P status of agricultural soils (McLaughhlin et al., 2011). Phosphate rocks are often a major component of slow- or controlled-release fertilizers (Bolan et al., 1990; Goh et al., 1990; Reijnders, 2014). Unlike annual crops which need high levels of P over a short period of rapid growth, pastures (especially legumes) require sustained sparingly soluble P sources and hence, phosphate rocks have been used extensively in grass-clover fertilization in temperate regions (van Diest, 1981; Sinclair et al., 1993). The use of rock phosphates in combination with biostimulants such as phosphate solubilizing bacteria and arbuscular mycorrhizal (AM) fungi has been introduced and marketed as a low-cost and low-energy mechanism to promote the agronomic effectiveness of rock phosphate fertilizers (Richardson, 2001; Gyaneshwar et al., 2002).

Pasture plant species differ in their capacity to exploit limiting nutrients (Hill et al., 2006) and this can influence their relative abundance within a sward (Driscoll and Strong, 2017). Long-term application of fertilizers, including slow-release fertilizers, can influence soil microbial communities (Zhao et al., 2014; Pan et al., 2016; Xun et al., 2016), and this in turn may influence the capacity of plants to access nutrients. With increasing interest in and availablility of commercial microbial inoculants marketed as biostimulants there is a need to understand potential modes of action and predictability of their efficacy (Abbott et al., 2018). Therefore, a glasshouse experiment was conducted to assess the impact of a multispecies microbial biostimulant on soil microbial communities in rhizospheres of two annual pasture species in comparison with application of fertilizers of different elemental solubilities, especially P.

While there is potential to improve nutrient use efficiency in pastures with application of slow-release fertilizers (Smith et al., 2018), benefits of inclusion of microbial biostimulants within management systems is less predictable because of inconsistency in responses (Qiu et al., 2019). Management of mixed annual pasture swards in south-western Australia has potential to benefit from incorporation of biostimulants due to relatively low fertilizer input (Bolland et al., 2011; Weaver and Wong, 2011; Gourley et al., 2017) and dependence on microbial processes associated with decomposition of organic matter, symbiotic nitrogen fixation and activities of soil microbial communities including phosphate solubilizing bacteria (Hinsinger et al., 2015). Therefore, we chose two common pasture species (subterranean clover and annual ryegrass) to investigate the efficacy of a multispecies microbial biostimulant and P fertilizers that differed in their solubilities in a glasshouse experiment based on previous studies of the same multispecies microbial inoculant with wheat (Assainar et al., 2018, 2020). The hypotheses were: (i) a multispecies microbial biostimulant will alter the rhizosphere bacterial community associated with two pasture species with different rooting structures (an annual legume and an annual grass) and (ii) a fast-release soluble P fertilizer will decrease rhizosphere soil bacterial diversity to a greater extent than a slow-release P fertilizer. The specific aim of this experiment was to identify soil bacterial community responses to microbial inoculants that may have potential as biostimulants in order to contribute to the longer-term aim of understanding and predicting modes of action of microbial biostimulants.

## Materials and Methods

A glasshouse experiment was conducted to evaluate the effects of a multispecies microbial biostimulant, in comparison to a rock mineral and a chemical fertilizer which differed in P solubility, on soil bacterial communities in the rhizospheres of two annual pasture plants that are commonly grown in south-western Australia.

### Soil Sampling

The soil was collected at a depth of 0–10 cm from an annual pasture at The University of Western Australia’s farm Ridgefield, Pingelly, WA (S 32°30′23″, E 116°59′31″, 116°59′48.50″E). A grid 25 × 25 m was used to select the area where the soil was collected using a zig-zag pattern. The soil was air dried and stored in a cool area and additional soil was collected for assessing bulk density. For 10 replicate soil samples, soil was passed through a 2 mm sieve to remove larger rock and plant residue particles. The initial characteristics of the soil were: 13.6% clay, 12% silt, 76% sand, pH 5.45 in H_2_O, pH 4.93 in CaCl_2_, and electrical conductivity (EC) 163 μS/cm. Available nutrients were assessed to as: Colwell P 65.8 mg kg^–1^ soil, NO_3_^–^ 1.8 mg kg^–1^ soil and NH_4_^+^ 19.52 mg kg^–1^ soil with a bulk density of 1.24 g/cm^3^ using methods described by Rayment and Higginson (1992).

### Experimental Design

The soil was potted into non-draining plastic pots (2 kg soil per pot). Four treatments were applied: (i) no soil amendment (control), (ii) a multispecies microbial biostimulant (Mic), (iii) a rock mineral fertilizer (MnF) at a rate of 75 kg ha^–1^ (∼5.6 kg P ha^–1^), and (iv) a chemical fertilizer (CF) at a rate of 43 kg ha^–1^ (∼5.6 kg P ha^–1^). There were two pasture species, subterranean clover (*Trifolium subterraneum* L. cv. “Dalkeith”) and Wimmera ryegrass (*Lolium rigidum* Gaudin), with four replicate pots of each treatment. Plants were harvested after 10 weeks.

The microbial inoculant used as a biostimulant consisted of a proprietary combination of various bacteria and fungi applied at a rate of 1 g per pot mixed in the top 30 mm of soil. Its trade name is Ag Blend Plus (produced by Australian Mineral Fertilizers Pty Ltd.). According to the distributor, it was a talc-based formulation containing (per g) isolates of *Agrobacterium rhizogenes* (1 × 10^9^), *Azotobacter* spp. (1.2 × 10^9^*), Azospirillum brasilense* (1.1 × 10^9^), *Bacillus subtilis* (112 × 10^9^), *Pseudomonas fluorescens* (2.3 × 10^9^), *Streptomyces* spp. (1 × 10^9^), *Trichoderma harzianum* (8 × 10^9^), and *Rhizophagus irregularis* (75 spores) (see also Assainar et al., 2018, 2020).

The composition of the fertilizers is listed in Table 1. The water solubility of P (assessed according to Chien, 1993) was 821 mg kg^–1^ in the chemical fertilizer and 657 mg kg^–1^ in the rock mineral fertilizer. The rock mineral fertilizer (from Australian Mineral Fertilizers Pty Ltd. called NPK Crop Plus) was a poorly soluble fertilizer consisting of a proprietary combination of various fine mineral ores. The ores include micas, alkali feldspars, soft rock phosphate, iron man gypsum (a byproduct from mineral sand processing, containing gypsum, iron and manganese), dolomite, basalt, granite and crystalline silica, and are blended with sulfate of ammonia and sulfate of potash, manganese sulfate, copper sulfate, and zinc sulfate (Storer and Devlin, 2012). The chemical fertilizer was a relatively soluble fertilizer from Summit Fertilizers Australia called Gusto Gold. The fertilizer treatments were added to the soil surface 1 day prior sowing and mixed with the soil. The microbial biostimulant was added as a powder to the seeds before sowing.

**TABLE 1:** Characterization of the rock mineral fertilizer (Ag Blend Plus from Western Mineral Fertilizers Pty Ltd.) and chemical fertilizer (Gusto Gold from Summit Fertilizer Australia).

**Characteristics**	**Rock mineral fertilizer (Ag Blend Plus)**	**Chemical fertilizer (Gusto Gold)**
N%	7.5	10.2
P%	7.5	13.1
K%	4.5	12
Ca%	5	0
S%	8	7.2
Mg%	0.9	0
Fe%	2.6	0
Si%	6.7	0
Mn, mg/kg	4,000	0
Zn, mg/kg	430	1,300
Cu, mg/kg	430	900
B, mg/kg	17	0
Ni, mg/kg	30	0
Mo, mg/kg	1.5	0
Bulk density, g/cm^3^	1.1	1.1

**Data supplied by the manufacturers.**

Subterranean clover and Wimmera ryegrass (5 plants per pot) were grown in separate pots for 10 weeks. Water content for all pots was maintained at 70% of field capacity by regular monitoring and addition of water to weight. Plants were grown in a glasshouse at The University of Western Australia under ambient light with a temperature range of 18/10°C (day/night). At harvest, the plants were lifted from the pots and shaken gently to remove soil. Soil subsamples were taken from the rhizosphere for DNA analysis (Mickan et al., 2017). Shoots and roots were dried at 60°C for 72 h then weighed to assess dry weight (DW). Total N and P from plant tissue were determined using a Kjeldahl digest (Bremner and Mulvaney, 1982). Total N was determined using an ammonium N in green method Na-Nitroprusside, and total P was calculated using molybdenum blue colorimetry (Blakemore et al., 1972).

### Available N, Extracted P and Soil Acidity

Soil mineral N (NO_3_^–^) and exchangeable N (NH_4_^+^) were measured following extraction of 20 g soil with 80 mL 0.5 M K_2_SO_4_ and analysis of the extracts colorimetrically for exchangeable NH_4_^+^ using the salicylate–nitroprusside method (Searle, 1984) and NO_3_^–^ concentration using the hydrazine reduction method (Kempers and Luft, 1988) on an automated flow injection Skalar AutoAnalyser (San plus, Skalar Analytical, Netherlands). Extractable Collwell P was determined for air-dried soils in 0.5 M sodium bicarbonate solution at pH 8.5 using the colorimetric methods of Rayment and Higginson (1992). Air dried soils were used to determine soil pH and EC. To determine pH in water suspension, 5 g of air-dried soil was suspended in 25 mL deionized water (1:5) and shaken for 1 h. For pH in CaCl_2_, 5 g of air-dried soil was suspended in 0.01 M of CaCl_2_ (Rayment and Higginson, 1992).

### DNA Extraction and Sequencing

Sub-samples of rhizosphere soil were used to extract bacterial DNA. DNA was extracted using the MoBio Powersoil DNA isolation kit (Geneworks, Australia) and quantified prior to storage at −20°C. Polymerase chain reaction (PCR) was then performed to amplify bacterial 16S rRNA genes from the DNA samples using Golay barcoded primers and PCR conditions described previously (Mickan et al., 2018). The amplification of the target 16S rRNA genes followed (Mickan et al., 2018) using 27F and 519R bacterial primers (Caporaso et al., 2010; Mori et al., 2014) amended by the barcodes of Golay (Caporaso et al., 2012) with negative controls. DNA sequencing was performed on the MiSeq platform at the Australian Genome research facility, Paired-end reads were assembled by aligning the forward and reverse reads using PEAR (version 0.9.5) (Zhang et al., 2014).

### Bioinformatics

The primers were identified and trimmed. Trimmed sequences were processed using Quantitative Insights into Microbial Ecology (QIIME 1.8) (Caporaso et al., 2010). Usearch (version 8.0.1623; Edgar, 2010; Edgar et al., 2011) and UPARSE software. Using Usearch tools DNA sequences were quality-filtered, and full-length duplicate sequences were removed and sorted according to abundance, singletons or unique reads in the data set were subsequently discarded. Sequences were clustered according to a chimera that was filtered using the “rdp_gold” database as a reference. To obtain the number of reads in each operational taxonomic unit (OTU), the reads were mapped back to the OTUs with a minimum identity of 97%. QIIME taxonomy was assigned using the Greengenes database (version 13_8, Aug 2013; DeSantis et al., 2006).

### Statistics

The experiment was set up as a bi-factorial design with the first factor being: “soil amendment” (control, microbial biostimulant, mineral fertilizer, chemical fertilizer), and the second factor being: “pasture type” (subterranean clover, Wimmera ryegrass). The interaction between “soil amendment” with “pasture type” was assessed using an two way ANOVA within the R environment. Data were checked for normality as part of the statistical analysis. The significance of “soil amendment” and “pasture type” driving bacteria community was assessed with PERMANOVA using distance matrices (Adonis function) and square root-transformed OTU relative abundance data in the R environment. A canonical correspondence correlation analysis (CCA) was used to explore the relationship between bacterial taxa with “soil amendment” and “pasture type” at the 97% OTU level with soil chemical and plant growth data. The variance inflation factor (VIF) was calculated for multiple regression models using the R package Vegan version 2.3.0 (Oksanen et al., 2010) and was used to evaluate if the variables should be included in the subsequent CCA. We used the criterion VIF < 3 to select suitable variables in the best multiple regression models to remove strongly multicollinear variables (Yang et al., 2017). The treatment means were compared using least significant differences (LSD). The analyses were performed using R version 3.4.3 (R Core Development Team, 2015, Austria, 2017) and Vegan version 2.3.0 (Oksanen et al., 2010) and GenStat V.12.1.5.3.

## Results

### Plant Biomass

#### Shoot Biomass

**FIGURE 1:**
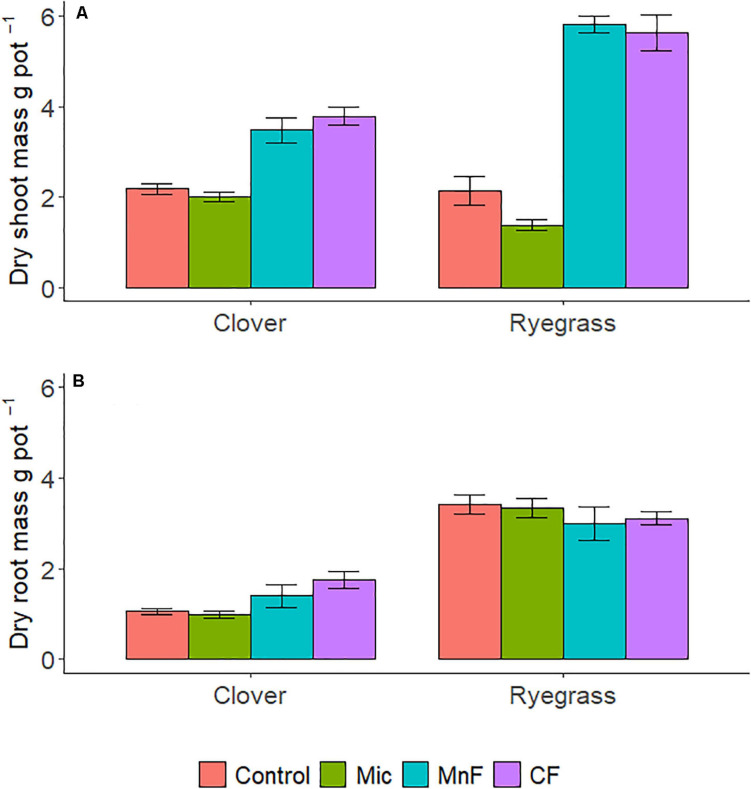
Dry shoot mass **(A)**, dry root mass **(B)** per pot for soil amendment treatments; Mic (microbial biostimulant), MnF (rock mineral fertilizer), and CF (chemical fertilizer), control (no amendment). Plant species were subterranean clover (*Trifolium subterraneum*) and Wimmera ryegrass (*Lolium rigidum*). Bars represent the mean, and error bars are the standard error of the mean (*n* = 4).

**FIGURE 2:**
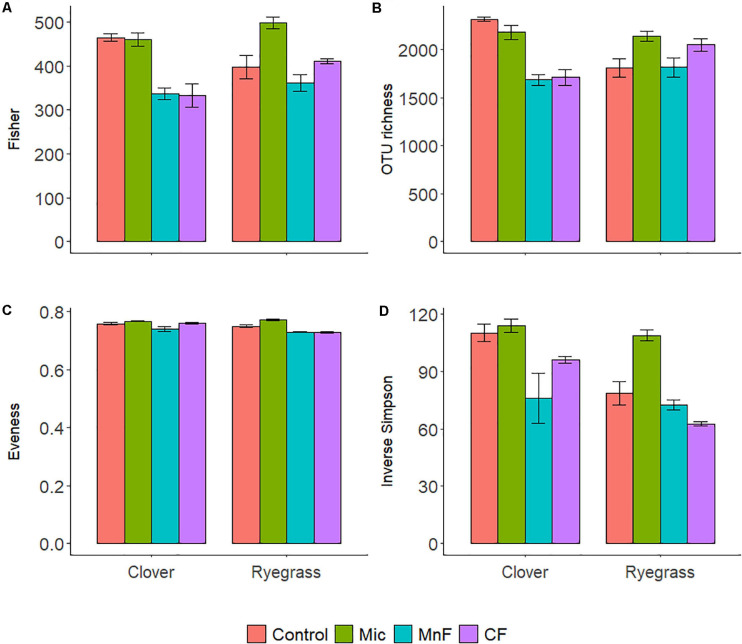
Alpha diversity calculators for soil bacteria at the 97% OTU level; Fisher’s **(A)**, richness **(B)**, Evenness **(C)**, Inverse Simpson **(D)**. For soil amendment treatments: Mic (microbial biostimulant), MnF (rock mineral fertilizer), and CF (chemical fertilizer), control (no amendment). Plant species were subterranean clover (*Trifolium subterraneum*) and Wimmera ryegrass (*Lolium rigidum*). Bars represent the mean, and error bars are the standard error of the mean (*n* = 4).

There were distinct changes in shoot biomass with both one way and two way ANOVA interactions for “soil amendment” and “pasture type” (Table 2 and Figure 1A). Where applicable, subsequent *post hoc* Tukey *T*-test within the plant species, subterranean clover dry shoot biomass was unaffected by seed inoculation with the microbial biostimulant. However, an increase in shoot biomass was observed for both rock mineral (*P* = 0.014) and chemical (*P* < 0.001) fertilizers compared to the untreated control soil (Figure 2A). Inoculation with the microbial biostimulant did not achieve the same level of shoot biomass as the rock mineral (*P* = 0.004) and chemical (*P* < 0.001) fertilizers. For ryegrass the microbial biostimulant did not increase shoot biomass (*P* = 0.355), but both the rock mineral (*P* < 0.001) and chemical (*P* < 0.001) fertilizers increased shoot biomass in comparison to the control soil (Table 2 and Figure 2A).

**TABLE 2:** Two-way ANOVA results showing *P*-values for dry shoot and root biomass per pot.

	**Treatment**	**Degrees of freedom**	**Sum of squares**	**Mean squares**	**F. model**	***P***
Shoot mass	Soil amendment	3	61.5	20.5	90.9	**< 0.001**
	Pasture	1	6.15	6.15	27.2	**< 0.001**
	Soil amendment: Pasture	3	12.3	4.12	18.2	**< 0.001**
	Residuals	24	5.41	0.22		
Root mass	Soil amendment	3	0.32	0.10	0.62	0.603
	Pasture	1	29.2	29.2	168.9	**< 0.001**
	Soil amendment: Pasture	3	1.61	0.53	3.10	0.045
	Residuals	24	4.15	0.17		

**Treatments consisted of “Soil amendment” and “Pasture.*” *Significant P-values are indicated in bold.**

#### Root Biomass

Root biomass was not influenced by soil amendment (*P* = 0.603), although pasture species (*P* < 0.001) and the interaction between “soil amendment” and “pasture type” were significant (*P* = 0.045) (Table 2 and Figure 1B).

### Soil Bacterial Community Assemblages

#### Alpha Diversity

There were distinct changes to all alpha diversity calculators for both one way and two-way ANOVA interactions for both “soil amendment” and “pasture type” (Table 3 and Figure 2). Subsequent *post hoc* Tukey *T*-test revealed no changes in Fisher’s alpha diversity for the microbial biostimulant applied for subterranean clover, but there was a reduction in Fisher’s alpha diversity with application of the rock mineral (*P* < 0.001) and chemical (*P* < 0.001) fertilizers (Figure 2A).

**TABLE 3:** Soil bacteria two-way ANOVA results showing *P*-values for alpha diversity calculators.

**Alpha diversity calculators**	**Treatment**	**Degrees of freedom**	**Sum of squares**	**Mean squares**	**F. model**	***P***
Shannon	Soil amendment	3	0.78	0.26	29.0	**< 0.001**
	Pasture	1	0.06	0.06	7.15	**0.013**
	Soil amendment: Pasture	3	0.09	0.03	3.34	**0.036**
	Residuals	24	0.22	0.01		
inverse Simpson	Soil amendment	3	6,718.7	2,239.5	16.9	**< 0.001**
	Pasture	1	2,728.3	2,728.2	20.7	**< 0.001**
	Soil amendment: Pasture	3	1,602.5	534.1	4.05	**0.018**
	Residuals	24	3,163.5	131.8		
Fisher	Soil amendment	3	82,957.0	27,652.5	22.4	**< 0.001**
	Pasture	1	2,625.0	2,625.1	2.13	0.158
	Soil amendment: Pasture	3	22,187.0	7,395.8	5.99	**0.003**
	Residuals	24	29,616.0	1,234.0		
Richness	Soil amendment	3	821,831.0	273,944.0	12.6	**< 0.001**
	Pasture	1	2,757.0	2,757.0	0.13	0.725
	Soil amendment: Pasture	3	771,849.0	257,283.0	11.8	**< 0.001**
	Residuals	24	521,137.0	21,714.0		
Evenness	Soil amendment	3	0.01	0.00	21.7	**< 0.001**
	Pasture	1	0.00	0.00	13.7	**0.001**
	Soil amendment: Pasture	3	0.00	0.00	5.54	**0.005**
	Residuals	24	0.00	0.00		

**Treatments consisted of “Soil amendment” and “Pasture.” Significant P-values are indicated in bold.**

In contrast, for ryegrass there was a significant increase in Fisher’s alpha diversity when the microbial biostimulant was applied (*P* = 0.009), but there were no effects of the rock mineral and chemical fertilizers (Figure 2A and Table 3). For subterranean clover, the microbial biostimulant had no effect on bacterial OTU richness, but it was decreased by both the rock mineral (*P* < 0.001) and chemical (*P* < 0.001) fertilizers (Figure 2B). Evenness was increased with application of the microbial biostimulant (*P* = 0.03) (Figure 2C) and the inverse Simpson index was affected by all treatments, “soil amendment” (*P* < 0.001), “pasture type” (*P* < 0.001), and there was an interaction between “soil amendment” and “pasture type” (*P* = 0.018) (Table 3 and Figure 2D). Addition of the microbial biostimulant, reduced inverse Simpson index for subterranean clover (*P* = 0.006), but increased it for ryegrass (*P* = 0.019) (Figure 2D).

#### Soil Bacteria Phylum Level Relative Abundance

**FIGURE 3:**
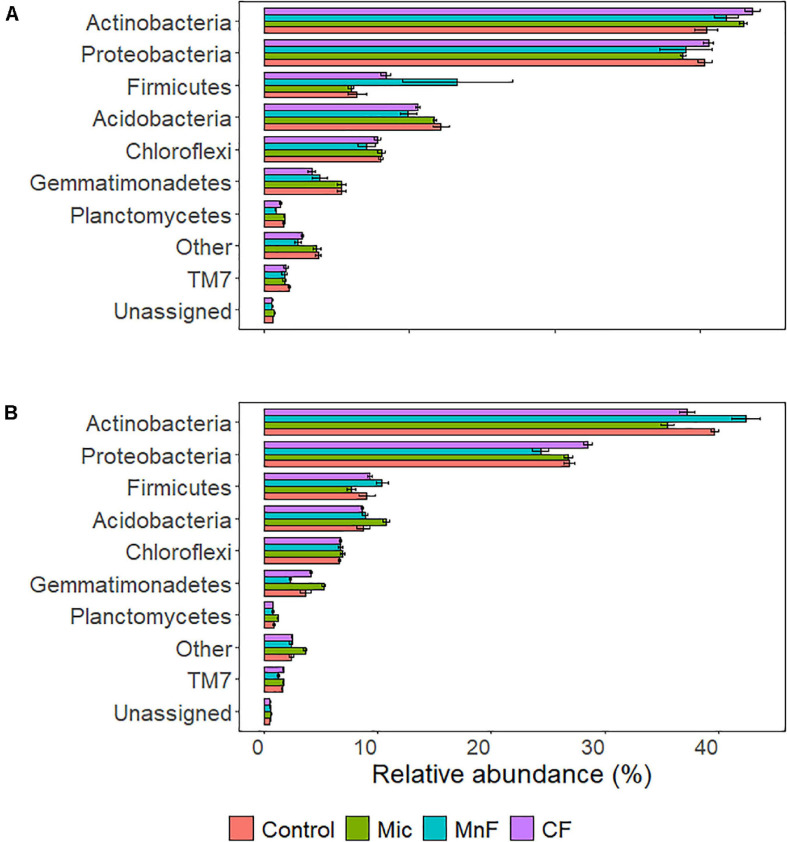
Soil bacterial relative abundance at the phylum level for plant species **(A)** subterranean clover (*Trifolium subterraneum*) and **(B)** Wimmera ryegrass (*Lolium rigidum*). For soil amendment treatments: Mic (microbial biostimulant), MnF (rock mineral fertilizer), and CF (chemical fertilizer), control (no amendment). Bars represent the mean, and error bars are the standard error of the mean (*n* = 4).

The relative abundance of bacteria at the phylum level in soil across all treatments was dominated by Actinobacteria (35%), Proteobacteria (27%), and to a lesser extent Acidobacteria (10%), Firmicutes (8%), Chloroflexi (7%), and Gemmatimonadetes (4%). There were changes to the relative abundance at the phylum level for “soil amendment” and “pasture type” with interactions between treatments (Table 4 and Figure 3).

**TABLE 4:** Soil bacteria two-way ANOVA results showing *P*-values fixed at Phylum resolution of relative abundance.

**Taxon**	**Treatment**	**Degrees of freedom**	**Sum of Squares**	**Mean squares**	**F. model**	***P***
Actinobacteria	Soil amendment	3	35.8	11.9	5.6	**0.005**
	Pasture	1	339.8	339.8	160.2	**< 0.001**
	Soil amendment: Pasture	3	97.6	32.6	15.3	**< 0.001**
	Residuals	24	50.9	2.1		
Proteobacteria	Soil amendment	3	35.3	11.8	5.1	**0.007**
	Pasture	1	75.3	75.3	32.7	**< 0.001**
	Soil amendment: Pasture	3	9.3	3.1	1.3	0.285
	Residuals	24	55.3	2.3		
Firmicutes	Soil amendment	3	114.1	38.0	4.8	**0.010**
	Pasture	1	3.0	3.0	0.4	0.548
	Soil amendment: Pasture	3	36.3	12.1	1.5	0.236
	Residuals	24	191.5	8.0		
Acidobacteria	Soil amendment	3	16.7	5.6	9.9	**< 0.001**
	Pasture	1	27.5	27.5	48.9	**< 0.001**
	Soil amendment: Pasture	3	7.9	2.6	4.7	**0.010**
	Residuals	24	13.5	0.6		
Chloroflexi	Soil amendment	3	1.7	0.6	1.8	0.180
	Pasture	1	7.8	7.8	25.1	**< 0.001**
	Soil amendment: Pasture	3	1.3	0.4	1.3	0.284
	Residuals	24	7.5	0.3		
Gemmatimonadetes	Soil amendment	3	22.5	7.5	17.8	**< 0.001**
	Pasture	1	3.0	3.0	7.3	**0.013**
	Soil amendment: Pasture	3	8.4	2.8	6.7	**0.002**
	Residuals	24	10.1	0.4		
Planctomycetes	Soil amendment	3	1.2	0.4	27.7	**< 0.001**
	Pasture	1	0.5	0.5	37.8	**<0.001**
	Soil amendment: Pasture	3	0.2	0.1	5.2	**0.006**
	Residuals	24	0.3	0.0		

**Treatments consisted of “Soil amendment” and “Pasture.” Significant P-values are indicated in bold.**

For subterranean clover, the relative abundance of Actinobacteria was not affected by inoculation with the microbial biostimulant (*P* = 0.251), the rock mineral fertilizer (*P* = 0.869) or the chemical fertilizer (*P* = 0.075) (Figure 3A). For ryegrass, the relative abundance of Actinobacteria decreased with application of the microbial biostimulant (*P* = 0.008) but increased in soil amended with the rock mineral fertilizer (*P* < 0.001) and was unaffected by the chemical fertilizer (*P* = 0.673) (Figure 3B).

For subterranean clover, the relative abundance of Proteobacteria was unaffected by the microbial biostimulant (*P* = 0.852), rock mineral fertilizer (*P* = 0.914) and chemical fertilizer (*P* = 0.999) (Figure 3A). There were no differences in relative abundance of Proteobacteria following application of the microbial biostimulant compared with the rock mineral (*P* = 0.999) and chemical (*P* = 0.728) fertilizers (Figure 3A). For ryegrass, the relative abundance of Proteobacteria was not affected by any treatment (Figure 3B). For the Firmicutes, there were minor changes in relative abundance associated with soil amendment (*P* = 0.009), but there was no effect of pasture species (*P* = 0.541) and there were no interactions between these treatments (Table 4).

For subterranean clover, the relative abundance of Acidobacteria was unaffected by the microbial biostimulant (*P* = 0.981), decreased with the rock mineral fertilizer (*P* = 0.005), and was unaffected by the chemical fertilizer (*P* = 0.095) (Figure 3A). The microbial biostimulant increased the relative abundance of Acidobacteria compared to the rock mineral fertilizer (*P* = 0.042) but not compared to the chemical fertilizer (*P* = 0.413) (Figure 3A). For ryegrass, the relative abundance of Acidobacteria increased with application of the microbial biostimulant (*P* = 0.017) but was not affected by the mineral fertilizer (*P* = 0.991) or the chemical fertilizer (*P* = 0.991) (Figure 3B). The microbial biostimulant increased the relative abundance of Acidobacteria under ryegrass compared with both the rock mineral (*P* = 0.034) and chemical (*P* = 0.011) fertilizers (Figure 3B).

The relative abundance of Chloroflexi was not affected by soil amendment (*P* = 0.180) but was affected by pasture species (*P* < 0.001), with no interactions between these treatments (Table 4 and Figure 3). For subterranean clover, the relative abundance of Gemmatimonadetes was not influenced by the microbial biostimulant (*P* = 0.991) or the rock mineral fertilizer (*P* = 0.058) but decreased with the chemical fertilizer (*P* = 0.004) (Figure 3A). The microbial biostimulant increased the relative abundance of Gemmatimonadetes compared to the mineral fertilizer (*P* = 0.004) but was unaffected by the chemical fertilizer (*P* = 0.413) (Figure 3A). For ryegrass, the relative abundance of Gemmatimonadetes increased with the microbial biostimulant (*P* = 0.035) but was unaffected by either the rock mineral (*P* = 0.102) or chemical (*P* = 0.971) fertilizer (Figure 3B). The microbial biostimulant increased the relative abundance of Gemmatimonadetes compared to the rock mineral fertilizer (*P* < 0.001) but not the chemical fertilizer (*P* = 0.249) (Figure 3B).

For subterranean clover, the relative abundance of Planctomycetes was unaffected by the microbial biostimulant (*P* = 0.996), but it decreased with the rock mineral fertilizer (*P* < 0.001) and was unaffected by the chemical fertilizer (*P* = 0.250) (Figure 3A). The microbial biostimulant increased the relative abundance of Planctomycetes compared to the rock mineral fertilizer (*P* < 0.001) but not the chemical fertilizer (*P* = 0.060) (Figure 3A). For ryegrass, the relative abundance of Planctomycetes increased with application of the microbial biostimulant (*P* = 0.013) but not the rock mineral (*P* = 0.898) or chemical (*P* = 0.751) fertilizers (Figure 3B). The microbial biostimulant increase the relative abundance of Planctomycetes compared to the rock mineral fertilizer (*P* = 0.001) but not the chemical fertilizer (*P* < 0.001) (Figure 3B).

#### Beta Diversity OTU Level Community Analysis

**FIGURE 4:**
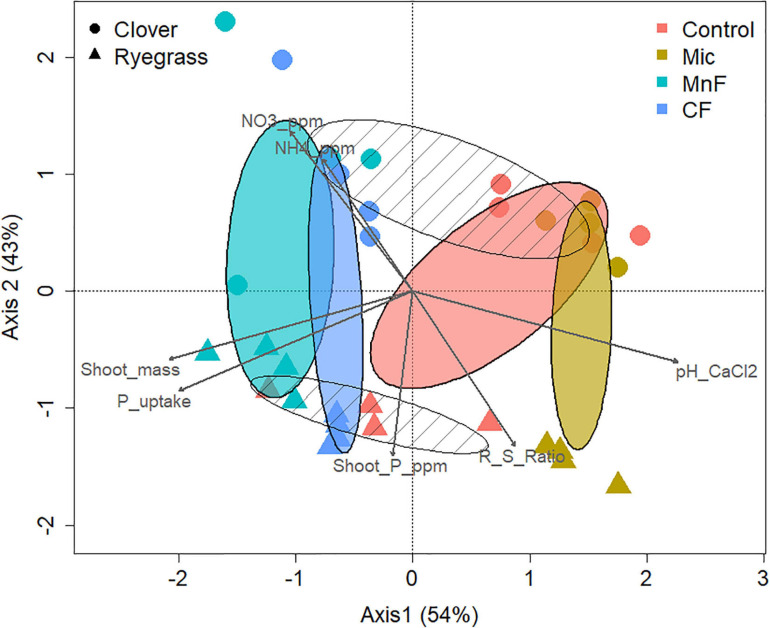
Canonical correspondence analysis of rhizosphere bacterial OTUs (97%) for soil amendment treatments: Mic (microbial biostimulant), MnF (rock mineral fertilizer), and CF (chemical fertilizer), control (no amendment). Plant species were clover (*Trifolium subterraneum*) and Wimmera ryegrass (*Lolium rigidum*). Shaded ellipses represent 95% confidence intervals of the soil amendment treatments, with hatched ellipses displaying plant species clusters.

To investigate the effects of the two pasture species and the form of fertilizer applied on the composition of the soil bacterial community at the 97% OTU level, a canonical correspondence analysis (CCA) was performed to determine which significantly correlated environmental variables with a VIF score < 3 (pH, NO^3–^–N, NH^4+^–N, shoot mass, P uptake, P concentration) best explained changes in bacterial community composition as assessed by a variation in inflation factor (Figure 4). Further analysis of community composition by PERMANOVA indicated significant community separation due to soil amendment (*P* < 0.001), pasture type *(P* < 0.001) and the interaction of soil amendment × pasture type (*P* < 0.002) (Table 5). The largest separation of OTU occurred along axis 1 (Figure 4) where there was a distinct clustering for the control samples that clearly separated from the soil amendments, with the microbial biostimulant clustering on the side most distant to the mineral and chemical fertilizer treatments. There was a distinct treatment effect on the bacterial community composition, with the communities from the untreated soil and soil treated with fertilizers clearly separating along axis 1 whilst the pasture species treatments separated along axis 2 as distinct communities (Figure 4).

**TABLE 5:** Soil bacterial community analysis by PERMANOVA results based on 97% similarity OTU abundance data (square root transformed), using 999 permutations.

**Treatment**	**Degrees of freedom**	**Sum of squares**	**Mean squares**	**F. model**	***R*^2^**	***P***
Soil amendments	3	0.296	0.098	4.88	0.253	**0.001**
Pasture	1	0.166	0.166	8.23	0.142	**0.001**
Soil amendment: Pasture	3	0.219	0.073	3.60	0.187	**0.002**
Residuals	24	0.486	0.020	0.41		
Total	31	1.169	1			

**Treatments consisted of “Soil amendment” and “Pasture.” Significant P-values are indicated in bold.**

## Discussion

Shoot dry weight was unaffected by inoculation with the microbial biostimulant and did not reach the levels achieved with either the rock mineral or conventional fertilizers, aligning with trends reported previously (e.g., Assainar et al., 2018, 2020). Root dry weight was less influenced by soil amendment, although there are morphological differences among these pasture species (Gilbert and Robson, 1984; Reid et al., 2015; Guy et al., 2018). This is consistant with previous studies showing a greater capacity for ryegrass to access soil P than subterranean clover (Barrow, 1975). This related to the greater area surface area of annual ryegrass roots compared with subterranean clover, and to its ability to decrease P concentration at the root surface to lower threshold concentration level (Barrow, 1975), which differ among plant species (Barber, 1980). These responses can also be related to root hair morphology, which can be longer and more dense when plants are growing in P-deficient soil (Foehse and Jungk, 1983; Bates and Lynch, 1996; Gahoonia and Nielsen, 1997; Ma et al., 2001). Nevertheless, there was no difference between the effect of the rock mineral and chemical fertilizers on root and shoot dry weight, probably reflecting the relatively high initial level P in this agricultural soil.

Although the two commercial fertilizers used in this study differed in their P solubilities, no difference in soil P concentration was measured for subterranean clover and annual ryegrass at the end of the experiment. Soil pH that can affect the solubility and availability of P from fertilizers in soil either directly (Hinsingher and Gilkes, 1996; Manning, 2008) or indirectly by decreasing the exudation of carbohydrates from roots (Graham et al., 1981).

Inoculation with the microbial biostimulant induced plant dependent responses to the diversity indices of soil bacteria including alpha diversity of bacterial OTUs richness (Shannon, Fisher, Richness, and Evenness). Some decreases in alpha diversity indices were similar to those reported previously by Assainar et al. (2020), and in soils with higher fertilizer inputs (Dai et al., 2018). In our experiment, most alpha diversity indices were less influenced by the microbial biostimulant in soil where subterranean clover was grown, and more influenced in soil where annual ryegrass was grown. However, decreases in alpha diversity observed were less consistent; some conventional fertilizers and microbial inoculants lead do increases in alpha diversity calculators (Assainar et al., 2018). Incorporation of composts applied to agricultural soil can also lead to substantial increases in alpha diversity calculators (Mickan et al., 2018), especially toward the latter phase of the plant growth cycle for organic material consisting of microbial residues (Zarezadeh et al., 2019).

**FIGURE 5:**
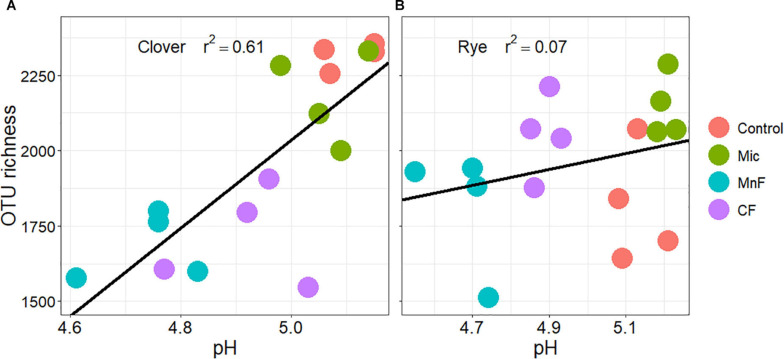
Correlation regression between OTU richness (97%), with soil pH for soil amendment treatments: Mic (microbial biostimulant), MnF (rock mineral fertilizer), and CF (chemical fertilizer), control (no amendment). Plant species were **(A)** subterranean clover (*Trifolium subterraneum*) and **(B)** Wimmera ryegrass (*Lolium rigidum*).

Both soil amendment and plant were major drivers of bacterial community structure in our study. The observed richness of OTUs (97%) was lower when the chemical fertilizer was applied to soil sown with subterranean clover than with annual ryegrass, but there was no effect of the microbial biostimulant. There was a highly significant correlation between soil bacterial OTU richness and soil pH for subterranean clover (*R*^2^ = 0.61) but no correlation for annual ryegrass (*R*^2^ = 0.07) (Figure 5). This plant dependent response could indicate direct relationships between soil bacterial community composition and plant-induced changes in soil pH, especially in the rhizosphere (Hinsinger et al., 2003). Alterations in soil pH may also be associated with microbial activity responsible for plant access to less available forms of P by reducing soil pH via production of organic acids (Marschner et al., 2011; Lagos et al., 2016). Community assemblages of soil bacteria have been demonstrated to be correlated with soil pH (O’Brien et al., 2019). Over longer time frames, the addition of fertilizer can also have a direct influence on soil bacteria by altering pH, with implications on nutrient cycling and P availability (Zhang et al., 2017). Whilst short term responses of bacterial communities can be associated with differences in plant species composition, this can be greater than those in response to fertilizer application (Bardgett et al., 1999). Our study demonstrates that a short-term response to both fertilizer and plant species can be related to either a direct influence of fertilizer or to indirect influences associated with soil microbial processes.

## Conclusion

The mechanisms contributing to an influence of plant species on soil bacterial community structure were highlighted in this study where seeds of two pasture plant species were inoculated with a multispecies microbial biostimulant. Although there was no beneficial effect of the microbial biostimulant on plant growth, there were significant influences on the soil bacterial community. The potential for interactions between biostimulants and the soil bacterial community provides scope for selection of plant-specific bacterial biostimulants in relation to either direct (fertilizer) and indirect (bacterial) localized changes to soil pH which could contribute to dissolution of poorly soluble forms of fertilizer, especially P fertilizers. The two commercial fertilizers investigated here differed in their P solubility and shoot biomass of subterranean clover and annual ryegrass both responded to their application in this agricultural soil. Fertilizers which varied in P solubility were associated with plant species dependent changes in naturally occurring soil bacterial communities. Further investigation could involve consideration of effects on soil microbial communities when selecting optimum rates of fertilizer, especially those that include poorly soluble P sources. Fertilizer application to mixed pasture communities should support soil microbial diversity and function involved in mineral dissolution processes at rates that meet plant requirements. Thus, further studies could consider the impacts of microbial biostimulants on soil bacterial communities, even when there are no plant growth responses to inoculation.

## Data Availability Statement

The data presented in the study are deposited in the NCBI database repository, accession number PRJNA638485.

## Author Contributions

AA and ZS conceived the original experimental design. AA conducted the experiment. BM initiated and completed the molecular analysis and led the interpretation of the data. All authors contributed to data interpretation and scientific writing.

## Conflict of Interest

The authors declare that the research was conducted in the absence of any commercial or financial relationships that could be construed as a potential conflict of interest.
